# Optimistic framing increases responsible investment of investment professionals

**DOI:** 10.1038/s41598-023-50965-w

**Published:** 2024-01-05

**Authors:** Dan Daugaard, Danielle Kent, Maroš Servátka, Lyla Zhang

**Affiliations:** 1 University of Tasmania, Hobart, Australia; 2 Discipline of Finance, University of Sydney Business School, The University of Sydney, Sydney, Australia; 3 MQBS Experimental Economics Laboratory, Department of Economics, Macquarie Business School, Macquarie University, North Ryde, Australia; 4 University of Economics in Bratislava, Bratislava, Slovakia

**Keywords:** Climate-change mitigation, Human behaviour

## Abstract

The global warming crisis is unlikely to abate while the world continues to collectively fund the extraction and burning of fossil fuels. Carbon divestment is urgently needed to ward off the impending climate emergency. Yet responsible investments still only account for a modest share of global assets. We conduct an incentivized artefactual field experiment to test whether framing divestment as a social norm, communicating it by a person with perceived credibility and expertise (a messenger), and highlighting optimistic attributes bolster responsible investment. Our subjects are investment professionals who have significant influence over the allocation of funds. We provide evidence that optimistic framing increases responsible investment. Assuming a comparable effect size, the observed increase would represent a $3.6 trillion USD global shift in asset allocations.

## Introduction

The accelerating global warming is arguably the most pressing problem that humanity is currently facing. Despite hundreds of world-wide appeals, public protests, and government declarations, the existing initiatives to stop and reverse climate change have proven to be insufficient as scientists keep moving the Doomsday Clock, an estimation for the likelihood of a man-made global catastrophe, closer to midnight^1^. This crisis is unlikely to abate while the world continues to collectively fund the extraction and burning of fossil fuels. Even with some high-profile institutional investors publicly divesting their fossil fuel holdings, the value of global divestments is relatively small^2,3^. Support for fossil fuel companies continues through funding and subsidies from governments and investment banks^4–6^. A radical change in investment choices is necessary.

Expert commentors warn that only a “massive reallocation of capital” will prevent global warming^7^. Institutional investors are key to driving this change because of their size and influence. Yet the proportion of responsible investment assets under management globally is only 36%^8^. In addition to reducing the direct funding support for fossil fuel companies, divestment also shifts the public discourse in relation to the legitimacy, reputation, and viability of the industry^9,10^. Institutional investors play a particularly important role in carbon divestment because investment professionals have the most influence over the proportion of responsible investments. For example, in Europe, the investment market is dominated by institutional investors who as of 2020, account for 72% of all assets under management. Only 28% of assets were related to retail investment^11^. Growth in responsible investment, or more precisely, Environmental, Social, and Governance (ESG) oriented investments, is urgently needed to ward off the impending climate emergency. Limiting global warming to 1.5 °C, as per the Paris Climate Agreement, requires a complete net decarbonization of the world’s energy by the middle of this century^12^. Doomsday-type messaging, prevalent in media, does not appear to be shifting carbon divestment enough. Taking a different approach to diverting investments away from carbon assets is thus paramount. Moving away from doomsday-type messaging could be an avenue to achieving it.

### Can framing increase responsible investing?

Previous research has demonstrated that the way information is presented, or “framed,” can lead people to make vastly different decisions for the same choice set^13–15^. Framing is particularly influential in choices involving the evaluation of risk and uncertainty^16–18^. To the best of our knowledge, however, there is no prior (experimental) research on whether framing can motivate responsible investment. The existing research either proposes conjectures which have not been subjected to rigorous testing, or only considers a narrow range of messaging frames (i.e., gain versus loss messaging). Furthermore, some research on responsible investing includes private wealth investors but very little extends to institutional investors^19,20^. In contrast, we apply framing to investment professionals who represent the segment of capital markets with the greatest influence on capital flows. While one may expect experienced investment professionals to be impervious to framing, there is evidence that professionals can be influenced by framing and make similar judgments as untrained individuals^21–23^.

While a potentially limitless number of frames can be examined, the practical and time constraints of conducting an experiment with investment professionals required us to focus our attention on testing a smaller set of frames based on established theories and evidence. We test the impact of social norm, optimism, and messenger frames, all of which have been shown to shift behavior in other contexts. We conjecture these three frames are likely to influence responsible investment^24^.

Moving away from carbon assets requires a collective effort that becomes easier to achieve if taking the desired action is perceived to be a social norm^25–27^. Studies on social norms and group dynamics reveal how individuals within a group can develop shared beliefs, values, and behaviors over time, leading to the establishment of social norms that influence individual behavior^28–31^. Reducing ambiguity around what the socially appropriate choice is or providing information about other people’s behavior can impact decisions^32,33^. If there exists an underlying social norm, framing is capable of shifting decisions^34^. Our social norm frame employs a descriptive norm, i.e., what most people typically do, as distinct from an injunctive norm, i.e., the perception of what is socially permissible. Descriptive norms have been shown to be powerful in shaping behavior. For instance, in a littering study where the amount of litter on the beach was randomly manipulated, researchers found that people were significantly less likely to litter on relatively clean days compared to the heavily littered days. Descriptive norms can influence behavior even in the absence of any explicit injunctions or rules^35^.

A core feature of investment is uncertainty. When there is uncertainty around future outcomes, some people have a bias for optimism^36^. Optimism has also been shown to predict pro-environmental behavior, whereas helplessness can act as a barrier to pro-environmental behavior^37,38^. A person with an optimistic explanatory style describes bad events as temporary and good events as permanent^39^. In the Optimism condition, we encourage responsible investment by leveraging the natural bias towards optimism. We test whether highlighting the temporary nature of the pain from divesting and the permanency of the benefits associated with divestment increases responsible investment.

Regarding the messenger frame, decisions, and judgments can sometimes be influenced by persuasion and arguments^40^. A particularly effective technique is using a messenger who carries greater knowledge, experience, or expertise to deliver the content^41^. Messengers ordained with perceived authority can make a message more persuasive^42–44^. We test whether a carbon divestment statement delivered by a messenger with noted industry experience in finance encourages greater responsible investment.

We test our conjectures in an artefactual online experiment with investment professionals whose decisions are incentivized. The results provide evidence that the optimism frame with an emphasis on the transitory nature of costs and the permanency of future benefits, significantly increases responsible investment by 3.6%. We find the social norm and messenger frames to be ineffective.

### Relationship to moral judgments

Responsible investment requires investors to make evaluative judgments that involve a moral component. While our experiment was not specifically designed to test moral judgments about climate action or carbon divestment, findings from moral psychology shed light on factors contributing to the insufficient response. Earlier research indicates that a major obstacle to mitigating climate change is that global warming fails to activate moral judgments that lead to action^45,46^. The thinking processes involving moral judgment are typically fast and intuitive and visceral reactions are recognized as an important driver for moral judgment, where individuals have a strong and unexplainable feeling of what is right or wrong^ 47–51^. In contrast to moral judgments about issues such as terrorism or child trafficking, which are more likely to elicit action, responsible investment may not activate a visceral response as a wrong that demands to be righted. Further, there is no explicit moral transgression around responsible investing that requires action because there is no identifiable individual acting intentionally to harm another individual^52,53^. As a result, actions to mitigate climate change are easily delayed or not undertaken at all, even by individuals who believe climate change is a problem and see the benefits of acting.

Disastrous messaging around climate change does not appear to be shifting carbon divestment enough. There is evidence that it could even be counterproductive. Catastrophic information about the severity of global warming can threaten an individual’s beliefs that the world is orderly. Individuals may then defensively respond by disengaging or even dismissing information about global warming to maintain their original position^54^. Taking a different approach to diverting investments away from carbon assets is thus paramount. We compare and test three communication strategies that could influence the moral judgments of investment professionals around responsible investing which subsequently may also impact their clients’ investments.

## Experiment

To test whether framing carbon divestment as a social norm, highlighting optimistic attributes, or using a messenger influences the propensity to invest in ESG assets, we conduct an artefactual field experiment in which experienced investment professionals are incentivized to construct their preferred investment portfolios. The decision-making environment is controlled using financial incentives as the expected payoffs directly depend on the subject’s allocation decisions in a given scenario.

The four experimental conditions (Control, Social Norm, Optimism, and Messenger; implemented in a between-subjects design) vary only in the framing of a preamble to the investment task. The preambles contain similar information about the impact of climate change on portfolio risk, though their framing highlights different aspects of the message. Table 1 provides a summary of the frames. The full text of all preambles and subject instructions are available in the Supplementary Information.

**Table 1:** Frames used to motivate responsible investment.

Control The general message highlights the risks of continued investment in fossil fuels: “*International financial monitoring bodies warn global warming is now a major financial risk.*”
Social Norm Presents similar information to the Control condition, except that the information is framed as a descriptive norm in financial markets:* “Most investors are now realizing that”*
Optimism Introduces similar information to the Control condition, except that the message contrasts the temporary cost of divestment with more permanent benefits of low carbon emissions over time “*In exchange for the temporary pain is a permanent gain”* as regulatory disruptions continue to grow
Messenger The message is delivered by an identifiable person, Bob Litterman, Chairman of the Board of Trustees at Commonfund, who understands *“the externalities created by burning fossil fuels… and the desire to position the portfolio to be aligned with his company’s mission.”*

Our original design included another condition to test the effect of a Loss frame which emphasized the potential losses from not divesting. We were unable to collect the Loss frame data because the conference organizers unintentionally sent the subjects who were randomly selected to be in the Loss frame the link to the Optimism frame. The sample for the Optimism frame became larger as a result. The individual attributes across conditions are distributed similarly because of the individual-level random allocation (see the Supplementary Information for mean attributes such as age and years of experience across conditions).

### Investment task

After reading the information in the condition-specific frame, each participant constructed the preferred investment portfolio for each of the six scenarios with a two-year horizon by allocating a $100 endowment among four investment options. The four options are: A—conservative investment, B—conservative investment with ESG orientation, C—balanced investment, and D—balanced with ESG orientation. Options B and D are responsible investment options while Options A and C do not have an ESG orientation. The sum of investments in the four options needed to equal AUD 100. For options that subjects did not want to invest in, they could choose zero. The investment task was identical for every subject.

The six scenarios differ in the following attributes: sustainability charge in the first year, and volatility (Table 2). The different scenarios are systematically constructed to allow further insights into how strengthening moral sentiments of responsible investing affects the trade-off between returns and risks^55^. The six scenarios also allow for checks on internal consistency (e.g., that lower return/higher risk combinations are not preferred to higher return/lower risk options). To prevent an order effect, the scenarios are ordered randomly for each participant.

**Table 2:** Information on investment options for the six scenarios in the experiment.

Attributes	Options	Conservative		Balanced	
		A	B	C	D
Environmental, social and government (ESG)	ESG orientation	No	Yes	No	Yes
	Sustainability charge to in the 1st year Scenario 1	0%	1.5%	0%	2.25%
	Scenario 2	0%	3%	0%	2.25%
	Scenario 3	0%	3%	0%	4.5%
	Scenario 4	0%	1.5%	0%	2.25%
	Scenario 5	0%	1.5%	0%	2.25%
	Scenario 6	0%	1.5%	0%	2.25%
Performance	Average annual return for the past 3 years	3%	2%	4%	3%
	Expected annual return for the next 10 years	4%	4.5%	6%	6.75%
Volatility	Standard deviation				
	Scenarios 1,2,3	4%	3%	6%	4.5%
	Scenarios 4,5	4%	4.5%	6%	6.75%
	Scenarios 6	4%	4%	6%	6%

The return, volatility, and sustainability charges for the investment options are designed to be consistent with what the subjects encounter in financial markets at the time of the experiment^56–58^. Realistic returns and risk numbers are employed across both scenario years. The portfolio return expectations are modeled using a risk-free rate of 0%. The one-off sustainability charge imposed on the ESG options reflects the short-run costs of carbon divestment.

### Procedures

The experiment took place during a major industry conference in Australia, on 30 September 2020. The conference was held online because of the Covid pandemic. To avoid priming effects from other sessions of the conference, the experiment was scheduled for the first session. Subjects were randomly assigned to experimental conditions. The experimenters received a list of conference registrants (with registrants’ email and phone numbers) from the conference organizers three days before the conference. The experimenters randomized registrants into five conditions (including the Loss condition) with separate experimental condition links using the rand() function in Microsoft Excel. The randomized list, sorted by treatment condition, was sent back to the conference organizers who were responsible for inviting participants by email to participate in the experiment during the conference. At the commencement of the experiment, subjects were sent a condition-specific link to their personal email address and were invited to participate using the link. All conditions were conducted simultaneously. There was no possibility for a subject to participate in more than one condition. Subjects completed the experiment individually and were not permitted to communicate with each other during the experiment to maintain the privacy of their decisions.

To increase the likelihood that subjects read the preamble information before proceeding to the next page, the preamble page was timed so that the ‘next’ button did not appear until 60 s after the page had loaded. After 60 s, the instruction to “Please click ‘next’ only after you've had a chance to read the text thoroughly” appeared. To check that subjects did indeed read the information thoroughly, we measured how long subjects remained on the page. The minimum time was 62 s with the average being 110 s, i.e., 50 s longer than required by design.

Subject instructions specified that the experimenters would randomly select 50 subjects to be paid via bank transfer for their decisions. To prevent potential wealth and portfolio effects, the individual payments depended on the risk and return of one of their chosen investment allocations, randomly selected from the six scenarios. Since each of the six scenarios had an equal chance of being chosen and the participants did not know in advance which would be chosen, they were explicitly asked to think about each portfolio carefully. To determine the payment for the drawn portfolio, the two-year return from the selected $100 portfolio allocation was calculated using the corresponding attributes. Options B and D with an ESG orientation incurred an initial sustainability charge applied to the first year only; there was no charge in the second year. If a subject selected one or both of these options, the charge was forwarded on his/her behalf to the Natural Resources Defense Council which is a charity working to safeguard the Earth—its people, its plants and animals, and the natural systems. At the end of the experiment, subjects filled out a questionnaire that included items from the Revised Life Orientation Test to measure subjects’ personal orientation towards optimism^59^.

Subjects selected for payment were contacted via email to obtain their bank account details and the payment was made via bank transfer. A replacement was drawn if a subject did not respond to the payment email within two days. All the above information was common knowledge. The average payment for the 50 paid subjects was AUD 107.80.

In total, 468 experienced investment professionals, such as portfolio managers, financial planners, service providers, and executives participated in the study. Using investment professionals as subjects provides for a rigorous test of our conjectures in the sense the subjects are sophisticated investors who are trained to make calculated judgments based on market indicators and as such should be less susceptible to framing. Equally importantly, the professional subject pool increases the external validity of our findings with respect to formulating policy recommendations because investment professionals overseeing portfolio investment allocations have significant influence over ESG-oriented investments. From an economic point of view, investment professionals have significant influence over the allocation of funds to responsible investments, which can further multiply the observed effects.

The experimental protocol was approved by the Human Research Ethics Committee at Macquarie University, Australia. Informed consent was obtained from all subjects and all methods were carried out in accordance with the guidelines and regulations set out by the ethics committee. The experiment was programmed in Lime Survey software.

## Results

We excluded 133 subjects (29 in Control, 32 in Social Norm, 56 in Optimism, and 16 in the Messenger condition) who completed the experiment but had no intention of increasing their investment in ESG within the next 10 years, or who identified as support-service providers not overseeing investment decisions because they were not in our target population.  Note that individuals in our sample may be considering increasing their ESG investments because it aligns with their values but could also be purely profit-driven and expect relatively higher returns from ESG investments. The analyzed sample size for the experiment was 335 investment professionals, 76% of whom were males.

We first compare the average ESG allocations across the four conditions that we collected data for. Our results show that the Optimism condition yields the highest average ESG allocation (Table 3, Panel A). Then to understand how a person’s level of optimism may interact with our implemented framing of responsible investing we decompose the results by optimistic life orientation (Table 3, Panel B). We find that optimistic investment professionals respond to optimistic framing. We then compare ESG Conservative and ESG Balanced allocations separately and observe greater framing effects in balanced options. Regarding the question of how framing impacts the trade-offs between volatility and returns, we observe that greater marginal volatility has little effect on average ESG allocations whereas one-off sustainability charges were negatively associated with average ESG allocations.

**Table 3:** Average ESG investment across all six scenarios.

	Control	Social Norm	Optimism	Messenger
Panel A: All subjects				
% ESG investment (options B and D) (Standard deviation)	64.24 (32.81)	63.56 (36.45)	67.87 (33.01)	64.71 (31.44)
Observations (subjects) = 2010 (335)	516 (86)	336 (56)	786 (131)	372 (62)
One-sided t-test (column condition vs control)	–	*t* = 0.276 *p* = 0.391	*t* = − 1.949 *p* = 0.026	*t* = − 0.215 *p* = 0.415
Panel B: Subjects with a more optimistic life orientation				
% ESG investment (options B and D) (Standard deviation)	63.04 (33.18)	60.80 (37.56)	73.53 (31.96)	67.96 (30.90)
Observations (subjects) = 1356 (226)	348 (58)	240 (40)	486 (81)	282 (47)
One sided *t*-test (column condition vs control)	–	*t* = − 0.745 *p* = 0.228	*t* = − 4.572 *p* < 0.001	*t* = − 1.922 *p* = 0.028
Panel C: Conservative ESG Allocations				
Mean investment (Standard deviation)	20.50 (23.36)	18.34 (21.21)	17.91 (22.55)	17.93 (20.55)
Observations (subjects) = 2010 (335)	516 (86)	336 (56)	786 (131)	372 (62)
One sided *t*-test (column condition vs control)	–	*t* = 1.395 *p* = 0.082	*t* = 1.983 *p* = 0.024	*t* = 1.715 *p* = 0.043
Panel D: Balanced ESG allocations				
Mean investment (Standard deviation)	43.74 (31.92)	45.23 (34.91)	49.97 (34.41)	46.78 (32.88)
Observations (subjects) = 2010 (335)	516 (86)	336 (56)	786 (131)	372 (62)
One sided *t*-test (column condition vs control)	–	*t* = − 0.626 *p* = 0.266	*t* = − 3.335 *p* < 0.001	*t* = − 1.374 *p* = 0.085

As a robustness check, we repeat the analysis in Panel A to include individuals who had no intention of increasing their investment in ESG within the next 10 years. The p-values are: Control vs Social Norm (p = 0.419); Control vs Messenger (p = 0.495); and Control vs Optimism (p = 0.146).

### The optimism frame produces the highest ESG allocations on average

The ESG allocations (in %) across our four conditions are reported in Table 3, Panel A. The Optimism condition yields the highest average ESG allocation (67.87%) followed by the Messenger (64.71%), Control (64.24%), and Social Norm (63.56%) conditions. The difference in ESG allocations between the Optimism condition and the Control condition is statistically significant using a one-sided *t-*test, (*p* = 0.026), while there is no statistically significant difference between the Social Norm or Messenger conditions and the Control condition (*p* = 0.391 and *p* = 0.415, respectively). The effect size as measured by Cohen’s *d* is 0.11.

To ensure the larger sample size of the Optimism condition is not responsible for the statistical significance, we conduct a robustness check. After randomly reducing the Optimism condition sample by 50% to be comparable in size to the other conditions, the difference is still statistically significant using a one-sided *t*-test (*p* = 0.041). The result confirms that under the optimistic frame investment professionals allocate more capital to ESG options.

### Optimistic investment professionals respond to optimistic framing

In our examination of the relationship between optimism and the implemented frames (see Table 3, Panel B), we restrict our samples to the investors who scored average or above (11 or more out of 15; henceforth “more optimistic”) in the Revised Life Orientation Test items^59^. When we compare more optimistic investors across conditions, those in the Optimism condition invested 10 percentage points more compared with the Control condition. The difference is statistically significant using a one-sided *t-*test (*p* < 0.001; Cohen’s *d* = 0.32), providing evidence that more optimistic investors do positively respond to an optimistically framed message about portfolio risk stemming from climate change. These results are further confirmed with a random effects panel regression, with robust errors clustered at the individual level. Regression output is provided in the Supplementary Information. We also find a positive effect of the Messenger framing on more optimistic investors (*p* = 0.028), but a statistically insignificant effect of the Social Norm framing (*p* = 0.228).

We compare whether more optimistic investors respond to our respective frames more strongly than less optimistic investors. Of particular interest is the response to optimistic framing. We find that more optimistic investors allocated approximately 15 percentage points more on average (73.53%) than less optimistic investors in the Optimism condition (58.70%). The difference is statistically significant using a one-sided *t*-test (*p* < 0.001; Cohen’s *d* = 0.46).

A greater response from more optimistic investors was also observed in the Messenger condition. More optimistic investors allocated significantly more 67.96% on average compared to 54.52% by less optimistic investors (*p* < 0.001 one-sided *t*-test; Cohen’s *d* = 0.29). In the Social Norm condition, more optimistic investors allocated 60.80% on average, while less optimistic investors allocated significantly more 70.47%, using a one-sided *t-*test (*p* = 0.010; Cohen’s *d* = 0.27).

To evaluate whether optimists choose a higher ESG allocation in general, we compare the average allocation of more optimistic investors (63.04%) and less optimistic investors (66.72%) in the Control condition. The difference is not statistically significant using a one-sided *t*-test (*p* = 0.113), implying that the changes in investment behavior are driven by the respective frames rather than by the more/less optimistic life orientation.

### Framing is more effective with balanced allocations

We compare ESG Conservative and ESG Balanced allocations separately to observe whether the framing effects differ between conservative and balanced options (see Table 3, Panels C and D). For balanced allocations, as with the combined ESG allocations, the Optimism condition yields significantly higher ESG allocations (49.97) compared to the Control condition (43.74). The result is significant using a one-sided *t*-test (*p* < 0.001; Cohen’s *d* = 0.30). The increase corresponds to a smaller, yet significant (*p* = 0.024; Cohen’s *d* = 0.11) decrease in responsible investment in the conservative ESG option for the Optimism condition compared to the Control condition. Similar effects are observed across the remaining conditions indicating that framing may encourage greater risk tolerance for responsible investment. Framing is therefore likely to be more effective in encouraging responsible investment with non-defensive assets.

### Investment professionals are sophisticated in their decision making

A common behavioral strategy enacted by investors is the diversification heuristic^60^. When investors are confused by the available choices, they sometimes adopt a naïve approach to diversification by simply spreading their investments evenly across the available choices. There were only 151 (5.4%) naively diversified portfolios out of 2808 portfolios in total across all conditions (see Fig. S3 in the Supplementary Information). This observation provides further evidence of the level of sophistication the investment professionals applied while participating in the experiment.

### One-off sustainability charges negatively impact ESG allocations

**Figure 1:**
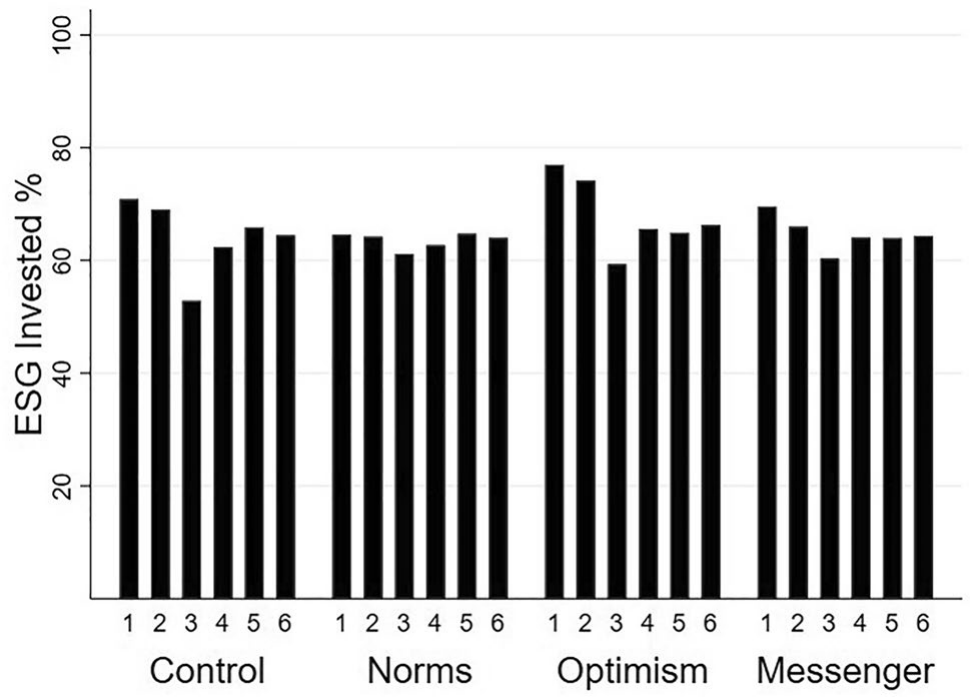
ESG (%) allocation for scenarios (1–6) by condition. The higher sustainability charge in Scenario 3 is associated with lower ESG investment. *N* = 335 subjects.

Figure 1 shows the ESG allocations across the six scenarios for each of the four conditions. The portfolio with the highest sustainability charges (Portfolio 3) has the lowest percentage of ESG invested across all conditions. The higher ESG allocation in Scenario 1 compared to Scenario 3 can be attributed to the lower ESG charge in Scenario 1 of 1.5% for the Conservative option and 2.25% for the Balanced option compared to the higher ESG charge of 3% and 4.5% in Scenario 3. The difference suggests an average 12.06% increase in preference for responsible investments in response to an average 1.7% fall in the cost of responsible investing. However, sensitivity varies across conditions. The Social Norms and Messenger conditions had less sensitivity than the Control and Optimism conditions.

### Higher marginal volatility does not negatively affect ESG allocations

We observe little impact of marginal increases in volatility risk on ESG allocations. Scenario 4 has higher volatility (standard deviation) for the ESG options than Scenario 6. The contrast between these scenarios therefore measures the impact that volatility has on ESG investment choices. Figure 1 shows the proportion allocated to ESG investments is slightly higher for Scenario 6 compared to Scenario 4 across the four conditions. If subjects were sensitive to the greater volatility risk associated with ESG we would observe substantially lower ESG investment in Scenario 4 compared to Scenario 6 and this is not the case. Investment professionals therefore appear to be insensitive to marginal increases in volatility risk when considering ESG allocations. One caveat is that differences in volatility among the six scenarios are modest and may not hold when dramatically different as might arise in highly volatile conditions.

## Discussion

The world is facing a climate crisis and innovative solutions are needed. Shifting capital away from fossil fuel industries and towards responsible investing is a key part of the solution. One major challenge for responsible investing is that it requires making evaluative judgments with a moral component. However, it appears that global warming fails to activate moral judgments that would lead to collective action^46,61^. In this article we report insights from an incentivized online experiment with investment professionals that point towards an effective communication strategy to increase responsible investment. The analyzed sample consists of individuals who stated their intention to increase their investment in ESG within the next 10 years and who are thus likely to be receptive to messages about climate change. We demonstrate that framing divestment decisions in a more optimistic orientation, with an emphasis on the transitory nature of costs and the permanency of future benefits, significantly increases responsible investment by 3.6%. With total professionally managed assets valued at USD $98.4 trillion globally, a comparable effect size would represent a USD $3.6 trillion shift in asset allocations^8^.

The presented experiment paves the way for future exploration of the mechanisms to foster greater engagement in responsible investing. Future research is necessary with respect to identifying the specific vehicles investment experts prefer for responsible investing and the appropriate methods for communicating the outcomes of responsible investments. The findings from our experiment contribute to understanding how the analytical facets of responsible investing could be enhanced by reframing the urgency of carbon divestment from doomsday to optimism.

### Supplementary Information

Supplementary Information 1.

Supplementary Information 2.

## Data Availability

Data is available in the Supplementary Information.
