# Supplementary material for: Optimistic framing increases responsible investment of investment professionals
Source: Sci Rep. 2024 Jan 5;14:583. doi: 10.1038/s41598-023-50965-w (PMC10770144; doi:10.1038/s41598-023-50965-w)
Supplement: Supplementary file 1 — Supplementary Information 1. [file 41598_2023_50965_MOESM1_ESM.pdf]

Supplementary Information: Optimistic framing increases responsible investment of investment professionals

Authors: Dan Daugaard, Danielle Kent*, Maroš Servátka, and Lyla Zhang.

Contents:

A. Experiment Instructions

B. Ethics Approval

C. Attributes (mean values) across conditions

D. Further Details on Experimental Procedures

E. Further Data Analysis

F. STATA Code

A. Experiment Instructions

Portfolio Choice Task

Please **do not start** until instructed. Instructions will be provided at 9:55am

Welcome!

Participating in this online task **contributes to your CE/CPD accreditation**, as proof of attendance at this session.

It should take approximately 20 minutes to complete.

You will be asked to read information about investment strategies and then allocate $100 between four investment options to create your most preferred portfolio.

In total, there will be six investment scenarios with different parameters, and you will create a portfolio for each of them.

After you have made your choices, a short questionnaire will follow.

Your choices are anonymous. Please do not share or discuss them with anyone during the experiment.

There are 24 questions in this survey.

Payment

We will randomly select 50 participants who will get paid for their decisions today. The payment will depend on the risk and return of one of your six investment portfolios.

The portfolio that is paid will be randomly selected out of the six. If you are selected for payment, we will contact you via email to obtain your bank account details and the payment will be made via bank transfer.

To determine your payment, we will calculate the return on an investment from your $100 portfolio.

Before we proceed to constructing portfolios, please read the following information relevant to the task. Further details about the portfolio construction task will be provided after this information.

Participant Consent

You are invited to participate in a study of economic decision making. The purpose of the experiment is to investigate how people make decisions in the environment described in the instructions.

Principal investigators: Dr Danielle Kent, Professor Maroš Servátka, Dr Lyla Zhang

If you decide to participate, the experiment will take approximately 20 minutes.

Any information or personal details gathered in the course of the study are confidential, except as required by law. Only the researchers will have access to the data and no individual will be identified in any publication of the results.

Participation in this study is entirely voluntary: you are not obliged to participate and if you decide to participate, you are free to withdraw at any time without having to give a reason and without consequence.

I have read and understand the information above and any questions I have asked have been answered to my satisfaction. I agree to participate in this research, knowing that I can withdraw from further participation in the research at any time without consequence.

Yes *

Please choose **only one** of the following:

- ○ Yes
- ○ No

Payment Details

In order to facilitate the payment, please provide your mobile number and email address.

The contact details you provide here will be used for the purposes of this experiment only.

What is your mobile phone number? *

Supplementary Information: Optimistic framing increases responsible investment of investment professionals

What is your work email address? *

How the returns are calculated

To determine the payment for the drawn portfolio, we will calculate the return on an investment from your $100 portfolio for **two years** using the corresponding attributes. (You can think of each day of the conference representing one year.)

After you have made your decisions, there will not be any further opportunities to change your portfolios, that is your portfolios will remain identical for 2 years. Note that there is no inflation in this experiment and the risk-free rate is 0%.

Options B and D with an ESG orientation will incur an initial sustainability charge that will be applied to the **first year only**, and not the second year. If you select one or both of these options, the charge will be forwarded on your behalf to the Natural Resources Defence Council who are a charity working to safeguard the Earth - its people, its plants and animals, and the natural systems. (You can think of the ESG charge as your donation to this charity generated by your portfolio.) We will forward you the joint contribution receipt as part of the follow up survey after the conference.

[CONTROL Treatment]

Please read the following IMPORTANT information regarding the experiment

International financial monitoring bodies warn global warming is now a major financial risk. There would be impact on global portfolio holdings if appropriate incentives to reduce greenhouse gas emissions were instituted worldwide.

High carbon emission assets would become less valuable and the valuations of companies holding those assets would fall. As expectations of such policies being implemented grow, there is a greater risk of fossil fuel assets becoming stranded assets.

While the absolute size of those stranded assets in some investment portfolios may be small, there are tiny holdings of stranded assets in many portfolios, making it difficult and expensive to arrange to sell all those assets.

The investor’s decision therefore needs to consider the management of portfolio risk in the face of our understanding of the reality of the externalities created by burning fossil fuels, the likelihood that stranded assets would underperform, and the desire to position aligned portfolios with the investor’s mission.

Please take as much time as you need. You will be able to proceed to the next step after 60 seconds.

Please note that you can still take more time to read the provided information. Please click "next" only after you've had a chance to read the text thoroughly.

[NORMS Treatment]

Please read the following IMPORTANT information regarding the experiment

International financial monitoring bodies warn global warming is now a major financial risk. There would be impact on global portfolio holdings if appropriate incentives to reduce greenhouse gas emissions were instituted worldwide.

Most investors are now realising that high carbon emission assets would become less valuable and the valuations of companies holding those assets would fall. As expectations of such policies being implemented grow, there is a greater risk of fossil fuel assets becoming stranded assets.

While the absolute size of those stranded assets in some investment portfolios may be small, there are tiny holdings of stranded assets in many portfolios, making it difficult and expensive to arrange to sell all those assets at the same time as everyone else.

The investor's decision therefore needs to consider the management of portfolio risk in the face of our understanding of the reality of the externalities created by burning fossil fuels, the likelihood that stranded assets would underperform, and the desire to position aligned portfolios with the investor's mission.

Please take as much time as you need. You will be able to proceed to the next step after 60 seconds.

Please note that you can still take more time to read the provided information. Please click "next" only after you've had a chance to read the text thoroughly.

[OPTIMISM Treatment]

Please read the following IMPORTANT information regarding the experiment

International financial monitoring bodies warn global warming is now a major financial risk. There would be impact on global portfolio holdings if appropriate incentives to reduce greenhouse gas emissions were instituted worldwide.

Reducing portfolio holdings away from high carbon emission assets can be temporarily challenging. Because while the absolute size of those stranded assets in some portfolios may be small, there are tiny holdings of stranded assets in many of the portfolios, making it difficult and expensive to arrange to sell all those assets.

In exchange for the temporary pain is a permanent gain in returns going forward. Low carbon emission assets will become more valuable over time and the valuations of companies holding those assets will continue to rise. Because, as expectations of greenhouse gas regulatory disruptions continue to grow, there will be ongoing and growing likelihood of fossil fuel assets becoming stranded assets.

The investor's decision therefore needs to consider the management of portfolio risk in the face of our understanding of the reality of the externalities created by burning fossil fuels, the likelihood that stranded assets would underperform, and the desire to position aligned portfolios with the investor's mission.

Please take as much time as you need. You will be able to proceed to the next step after 60 seconds.

Please note that you can still take more time to read the provided information. Please click "next" only after you've had a chance to read the text thoroughly.

[MESSENGER Treatment]

Please read the following IMPORTANT information regarding the experiment

International financial monitoring bodies warn global warming is now a major financial risk. There would be impact on global portfolio holdings if appropriate incentives to reduce greenhouse gas emissions were instituted worldwide.

Two years ago Bob Litterman, Chairman of the Board of Trustees at Commonfund, asked himself what the impact on their portfolio holdings would be if appropriate incentives to reduce greenhouse gas emissions were instituted globally. The answer was obvious – high carbon emission assets would become less valuable and there would be a greater risk of fossil fuel assets becoming stranded assets.

He and his astute portfolio managers anticipated that as expectations of such policies being implemented grew, it would negatively impact the valuations of stranded assets. And while the absolute size of those stranded assets in his portfolio was small, there were tiny holdings of stranded assets in many of the portfolios that they invested in, making it difficult and expensive to arrange to sell all those assets.

In order to reduce the expense and complexity of selling those assets Bob Litterman decided to create the equivalent economic exposure by entering into an innovative financial instrument, a "stranded asset total return swap." The swap was a very simple contract between two counterparties, in this case with Deutsche Bank. Every three months one party pays the other depending on whether stranded assets have outperformed or underperformed the market. Bob Litterman therefore protected Commonfund portfolio against the risk created by these "stranded assets." It was a decision based on managing portfolio risk in the face of Bob and his portfolio managers' understanding of the reality of the externalities created by burning fossil fuels, the likelihood that stranded assets would underperform, and the desire to position the portfolio to be aligned with his company's mission.

Please take as much time as you need. You will be able to proceed to the next step after 60 seconds.

Please note that you can still take more time to read the provided information. Please click "next" only after you've had a chance to read the text thoroughly.

Constructing Six Portfolios

In the decision task that follows, you will be presented with six scenarios, one at a time. The scenarios were generated for this task and are not meant to reflect the existing market conditions.

For each of the scenarios you will construct your most preferred portfolio by allocating $100 among the four investment options, A, B, C, and D. Some of the attributes of these four options might differ between scenarios.

You will create your portfolio by indicating the amount you would like to put on each option.

The sum of investments into the four options **must be equal to $100**.

Please **choose 0** for options you do not want to invest in.

Please note that each of the six scenarios has an equal chance of being chosen and you do not know in advance which will be chosen, so think about each portfolio carefully.

**Attributes**		Conservative		Balanced	
		A	B	C	D
Environmental, social and government (ESG)	ESG orientation	No	Yes	No	Yes
	Sustainability Charge to You in the 1st year	0%	1.5%	0%	2.25%
Performance	Average annual return for the past 3 years	3%	2%	4%	3%
	Expected annual return for the next 10 years	4%	4.5%	6%	6.75%
Volatility	Standard deviation	4%	3%	6%	4.5%

*Please ensure the total is 100

	**A**	**B**	**C**	**D**
**Allocation**				

Please **choose 0** for options you do not want to invest in.

**Attributes**		Conservative		Balanced	
		A	B	C	D

Environmental, social and government (ESG)	ESG orientation	No	Yes	No	Yes
	Sustainability Charge to You in the 1st year	0%	3%	0%	2.25%
Performance	Average annual return for the past 3 years	3%	2%	4%	3%
	Expected annual return for the next 10 years	4%	4.5%	6%	6.75%
Volatility	Standard deviation	4%	3%	6%	4.5%

*Please ensure the total is 100

	A	B	C	D
**Allocation**				

Please **choose 0** for options you do not want to invest in.

**Attributes**		Conservative		Balanced	
		A	B	C	D
Environmental, social and government (ESG)	ESG orientation	No	Yes	No	Yes
	Sustainability Charge to You in the 1st year	0%	3%	0%	4.5%
Performance	Average annual return for the past 3 years	3%	2%	4%	3%
	Expected annual return for the next 10 years	4%	4.5%	6%	6.75%
Volatility	Standard deviation	4%	3%	6%	4.5%

*Please ensure the total is 100

	A	B	C	D
**Allocation**				

Please **choose 0** for options you do not want to invest in.

Supplementary Information: Optimistic framing increases responsible investment of investment professionals

Attributes		Conservative		Balanced	
		A	B	C	D
Environmental, social and government (ESG)	ESG orientation	No	Yes	No	Yes
	Sustainability Charge to You in the 1st year	0%	1.5%	0%	2.25%
Performance	Average annual return for the past 3 years	3%	2%	4%	3%
	Expected annual return for the next 10 years	4%	4.5%	6%	6.75%
Volatility	Standard deviation	4%	4.5%	6%	6.75%

* Please ensure the total is 100

	A	B	C	D
**Allocation**				

Please **choose 0** for options you do not want to invest in.

Attributes		Conservative		Balanced	
		A	B	C	D
Environmental, social and government (ESG)	ESG orientation	No	Yes	No	Yes
	Sustainability Charge to You in the 1st year	0%	1.5%	0%	2.25%
Performance	Average annual return for the past 3 years	3%	2%	4%	3%
	Expected annual return for the next 10 years	4%	4.5%	6%	6.75%
Volatility	Standard deviation	4%	4%	6%	6.75%

*Please ensure the total is 100

	A	B	C	D
**Allocation**				

Please **choose 0** for options you do not want to invest in.

Supplementary Information: Optimistic framing increases responsible investment of investment professionals

Attributes		Conservative		Balanced	
		A	B	C	D
Environmental, social and government (ESG)	ESG orientation	No	Yes	No	Yes
	Sustainability Charge to You in the 1st year	0%	1.5%	0%	2.25%
Performance	Average annual return for the past 3 years	3%	2%	4%	3%
	Expected annual return for the next 10 years	4%	4.5%	6%	6.75%
Volatility	Standard deviation	4%	4%	6%	6%

*Please ensure the total is 100

	**A**	**B**	**C**	**D**
**Allocation**				

Please **choose 0** for options you do not want to invest in.

Demographics

Which type of organizations do you work for? *

Choose one of the following answers
Please choose **only one** of the following:

- FP = Financial Planning
- FP-FO = Financial Planning / Family Office
- PI = Private Investor
- SP-ASSOC = Service Provider/ Association
- SP-CO = Service Provider/ Consulting
- SP-ED = Service Provider/ Education
- SP-FM = Service Provider/ Funds Management
- SP-FM-MTM = Service Provider/ Funds Management (Multi-manager)
- SP-MKTG = Service Provider/ Marketing/PR
- SP-PLAT = Service Provider/ Trading platform
- SP-RSH = Service Provider/ Research House / Investment Consulting House
- SP-SF = Service Provider/ Super Fund
- SP-TRUSTCO = Service Provider/ Trust Company
- Other

What is your job position? *

Please choose….<br>v

- BUS = Business / Operational role
- P = Principal of the firm
- FP = Financial adviser
- PP = Paraplanner
- RSH = Research analyst
- IC = Investment Consultant
- ED = Teacher/Lecturer
- EXEC = C suite level
- S&M = Sales & Marketing
- INV = Investment role
- Other

Are you an Advocate or Practitioner?
*Please choose **only one** of the following:

○ An Advocate (Advocates champion specific funds to Practitioners for use in portfolios.)
○ A Practitioner (Practitioners have some influence over whether a fund is used or not.)
○ Neither

What is your gender? *

Please choose **only one** of the following:

○ Male
○ Female
○ Prefer not to answer

What was your age at your last birthday? *

*Only numbers may be entered in this field.

How long have you been working in the Finance industry? *

*Only numbers may be entered in this field.

This includes the time in different roles.

Please indicate the extent you agree with each of the following statements.

[1 = strongly disagree; 2 = disagree; 3 = neutral; 4 = agree; 5 = strongly agree]

*

Please choose the appropriate response for each item:

	1	2	3	4	5
**In uncertain times, I usually expect the best**					
**I'm always optimistic about my future**					
**I hardly ever expect things to go my way**					

Please indicate the extent you agree with each of the following statements.

[1 = strongly disagree; 2 = disagree; 3 = neutral; 4 = agree; 5 = strongly agree]

*Please choose the appropriate response for each item:

	1	2	3	4	5
**I expect the price of ESG investments to increase over time**					
**The environment is important to me**					
**I put a lot of thought into my investment decisions**					
**The regulatory risk of high carbon emission assets is high**					
**The regulatory risk of high carbon emission assets will increase**					

In the near future, I plan to increase the share of ESG investments in my personal portfolio.

*Check all that apply

- In a month
- In a year
- In 10 years
- I do not plan to increase my share of ESG investments

Was the amount of money offered enough for you to take the task seriously? *

- Yes
- No

What motivated your choices in the experiment?

Is there anything else you want to tell us?

Submit

Thanks for participating! Your participation is noted.

The overall results of this task will be presented on the second day of the Forum.

Supplementary Information: Optimistic framing increases responsible investment of investment professionals

****

B. Ethics Approval

Informed consent was obtained from all participants and the experiment was performed in accordance with Macquarie University Human Research Ethics Committee approval. ref. # 5201700434

C. Attributes (Mean values) across conditions

Variable	Control	Norms	Optimism	Messenger
LOT-R items average score	11.28	11.03	11.01	11.28
Average Age (years)	43.44	44.16	43.11	44.10
Prop of Females	0.27	0.26	0.19	0.37
Years of Experience	19.14	18.80	18.61	17.96

Table S1: Mean values of attributes across conditions.

D. Further Details on Experimental Procedures

The experiment was conducted online on September 30, 2020 as part of a portfolio Markets Summit, a continuing education event organized by the Portfolio Construction Forum (PCF) with a live hybrid program with delegates from across Australia and New Zealand. PCF is a specialist and independent investment continuing education body and accreditation and certification service. PCF provides a curriculum curated for the community of wealth management professionals. Participants who registered for this conference were finance professionals such as portfolio managers, financial planners, service providers, and executives.

Randomization

The experimenters received a list of conference registrants (with registrants' email and phone numbers) from the conference organizers 3 days before the conference. The experimenters randomized registrants into five conditions with separate experimental condition links using the *rand()* function in Microsoft Excel. The randomized list, sorted by treatment condition, was sent back to the conference organizers who were responsible for inviting participants by email to participate in the experiment during the conference.

The experiment

To avoid potential priming and confounding effects stemming from the conference program, the experiment took place in the first session of the conference. Participants were sent a treatment-specific link to their personal email address that they used to register for the conference. The decisions were elicited using the Lime Survey platform. The platform only allowed one submission from a single IP address and submissions were closed at the conclusion of the experiment.

Supplementary Information: Optimistic framing increases responsible investment of investment professionals

Participants were asked not to share or discuss their responses with anyone during the experiment. They were also instructed that the experimenters would randomly select 50 participants to get paid for their decisions. The payment depended on the risk and return of one of their six investment portfolios. The portfolio that was paid, was randomly selected out of the six. Since each of the six scenarios had an equal chance of being chosen and the participants did not know in advance which would be chosen, they were explicitly asked to think about each portfolio carefully.

To determine the payment for the drawn portfolio, the two-year return from the selected $100 portfolio allocation was calculated using the corresponding attributes. Options B and D with an ESG orientation incurred an initial sustainability charge applied to the first year only; there was no charge in the second year. If a participant selected one or both of these options, the charge was forwarded on his/her behalf to the Natural Resources Defense Council who are a charity working to safeguard the Earth - its people, its plants and animals, and the natural systems. All participants selected for payment were forwarded the joint contribution receipt. After the investment task, participants were given a questionnaire which included items from the Revised Life Orientation Test to measure the respondent's level of optimism and demographic questions.[1]

Participants selected for payment were contacted via email to obtain their bank account details and the payment was made via bank transfer. A replacement was drawn if a participant did not respond to the payment email within two days. All the above information was common knowledge.

Our final sample consists of 335 individuals: 76 percent males, 22 percent females and 2 percent identifying as other. The average age of individuals in our final sample was 44 years old, with an average of 18.34 years of experience. There were 424 participants who completed the experiment. We excluded 89 individuals who had no intention of increasing their investment in ESG within the next 10 years, or who identified as support-service providers that did not oversee investment decisions because they were not in our target population. 50 participants were randomly selected for payment, with an average payment of AUD $107.80.

Options and returns were based on typical market conservative and balanced options. Asset allocations for typical conservative and balanced options are demonstrated by Blackrock's Product Disclosure Statement[2]. The conservative investment option contains large exposures to less volatile asset classes such as fixed income and cash. In contrast, the balanced investment option holds greater allocations to the growth-oriented asset classes such as Australian and international equities. Investors typically select one option or a combination of the two depending on their risk attitudes and their expectation about future returns.

The identifiable person used as the messenger in the Messenger frame is Robert Litterman, Chairman of the Board of Trustees at Commonfund. His public position on climate change and responsible investing was used in our Messenger framing.

---

[1] The Revised Life Orientation Test (LOT-R) is a scale that measures how optimistic or pessimistic people feel about the future. See Scheier, M. F., Carver, C. S., & Bridges, M. W. (1994). Distinguishing optimism from neuroticism (and trait anxiety, self-mastery, and self-esteem): A re-evaluation of the Life Orientation Test. Journal of Personality and Social Psychology, 67(6), 1063-1078.

[2] BlackRock. (2009) *Balanced Funds Product Disclosure Statement*. Retrieved February 2022 from: https://wealth.mlc.com.au/content/dam/wealth/public/documents/fdd/PWA0822AU.pdf

Supplementary Information: Optimistic framing increases responsible investment of investment professionals

E. Further Analysis

Fig. S1 shows that the Optimism frame resulted in a 4-percentage point higher ESG allocation compared to the Control condition.

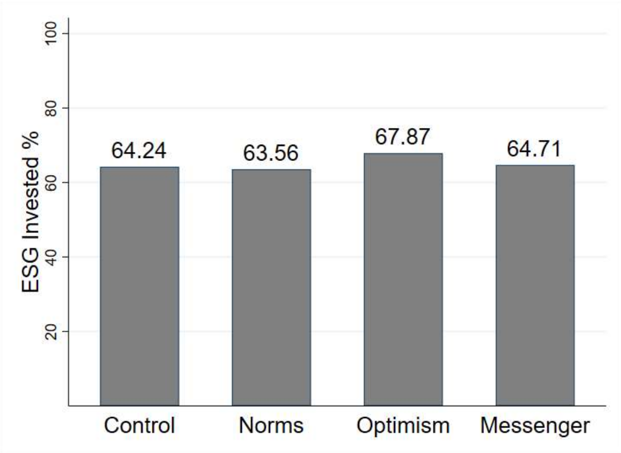

Fig. S1: ESG (%) allocation of finance professionals who intended to increase their investments in ESG in the future (responding either in a month, year or in 10 years) in each condition.
Notes: N=335 subjects; Control=86; Norms=56; Optimism=131; Messenger =62.

Effect Size: Cohen's d 0.11 (Control verses Optimism Condition)

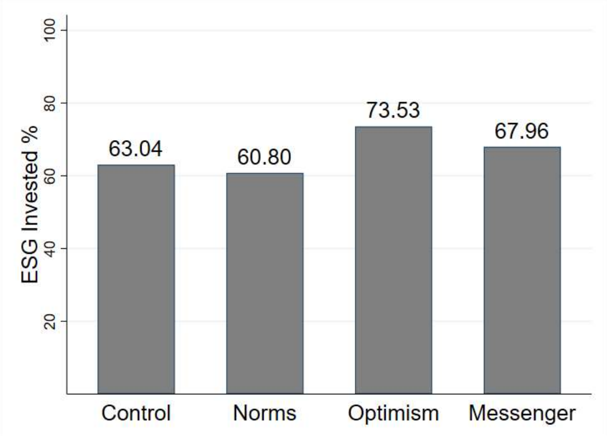

Fig. S2: ESG (%) allocated by investment professionals who had a more optimistic life orientation. Notes: N=226 subjects, Control=58; Norms=40; Optimism=81; Messenger =47.

Effect Size: Cohen's d 0.32 (Optimists in Control verses Optimists in Optimism Condition)

Supplementary Information: Optimistic framing increases responsible investment of investment professionals

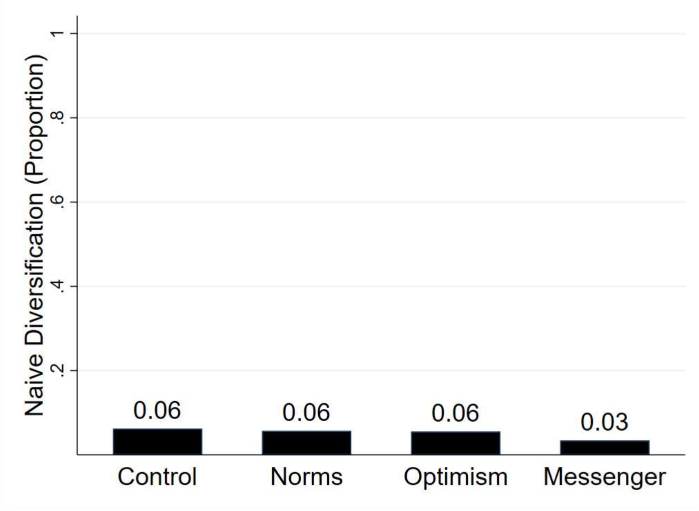

Fig. S3: Proportion of naively diversified portfolios by condition. Notes: 151 portfolios naively diversified across all treatments from a total of 2,808 portfolio allocations.

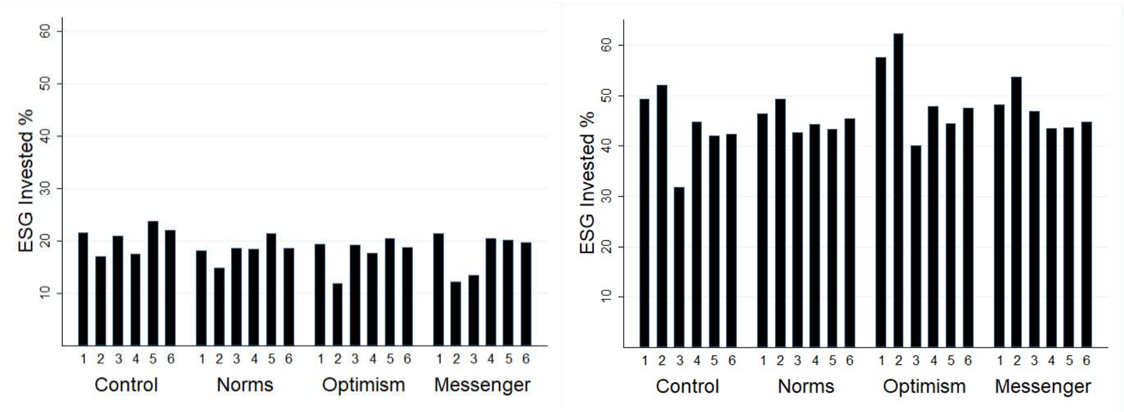

Fig. S4. Conservative ESG (%) allocation (left panel) and Balanced ESG (%) allocation (right panel) for Scenarios 1-6 by condition. These panels illustrate that investors are rebalancing the overall portfolio risk. This is most apparent by comparing the Conservative allocations with the Balanced allocations for Scenarios 4, 5 and 6. Note the risk on the Conservative ESG option decreases between Scenarios 4 and 5. If we ignore any trade-off between risk and responsible investing, we would expect to see a rebalancing towards the Conservative options from the Balanced options. This shift can be observed across four of the conditions (i.e., Control, Norms, Optimism and Messenger). In a similar way, we note the risk on the Balanced ESG option decreases between Scenario 5 and 6. Aside from risk versus responsible investing preferences, a rebalancing towards Balanced from Conservative options should occur. We see this anticipated shift across all four conditions.

Post Experiment Questionnaire responses

The following graphs illustrate the distribution of responses to the post experiment questionnaire questions relating to subject's attitudes and expectations.

Supplementary Information: Optimistic framing increases responsible investment of investment professionals

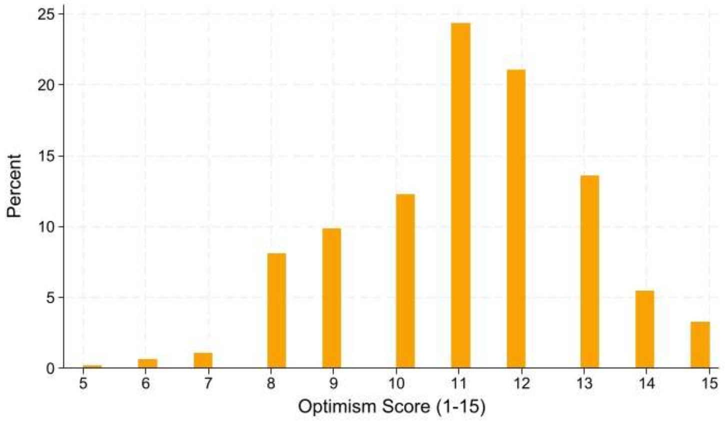

Fig S5. Distribution of total score of Life Orientation (LOT-R) Items

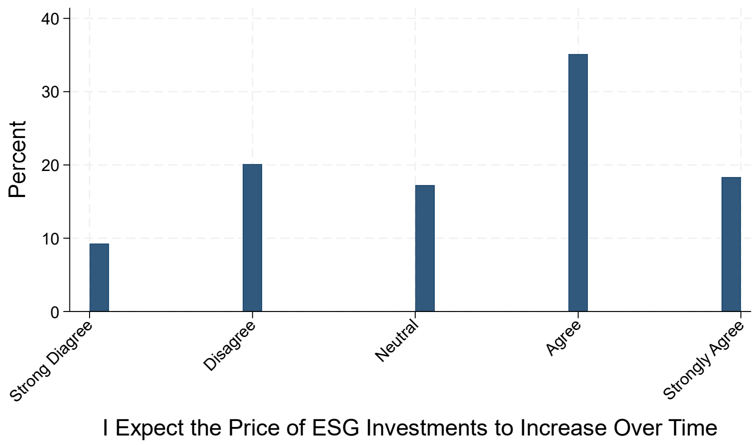

Fig. S6. Distribution of responses to "I expect the price of ESG investments to increase over time"

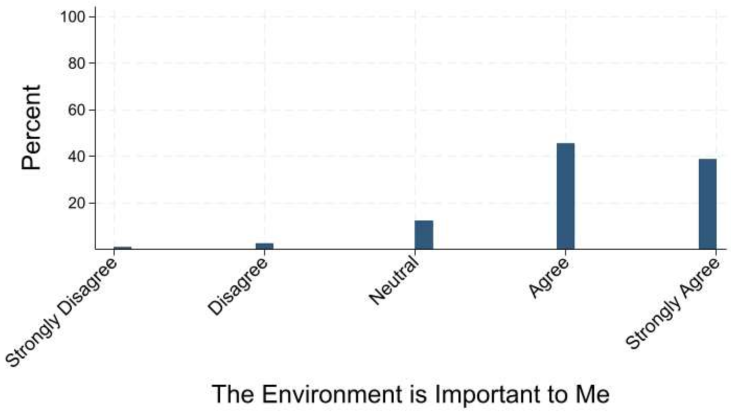

Fig. S7. Distribution of responses to "The environment is important to me"

Supplementary Information: Optimistic framing increases responsible investment of investment professionals

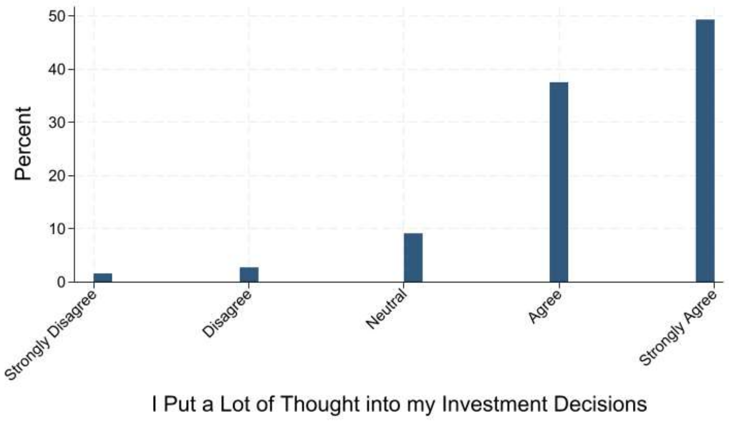

Fig. S8. Distribution of responses to "I put a lot of thought into my investment decisions"

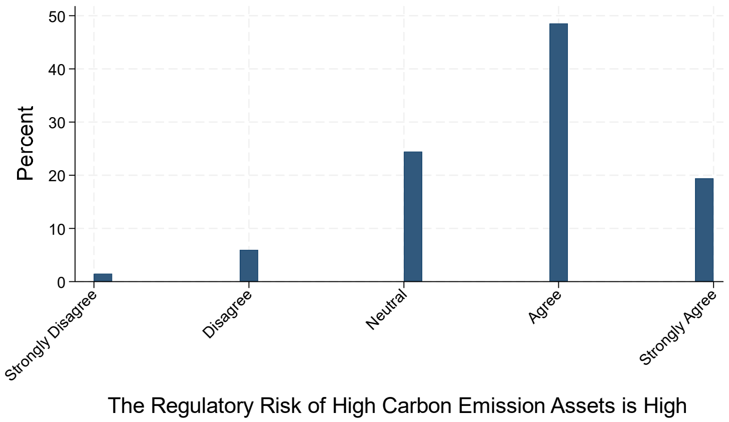

Fig. S9. Distribution of responses to "The regulatory risk of high carbon emission assets is high"

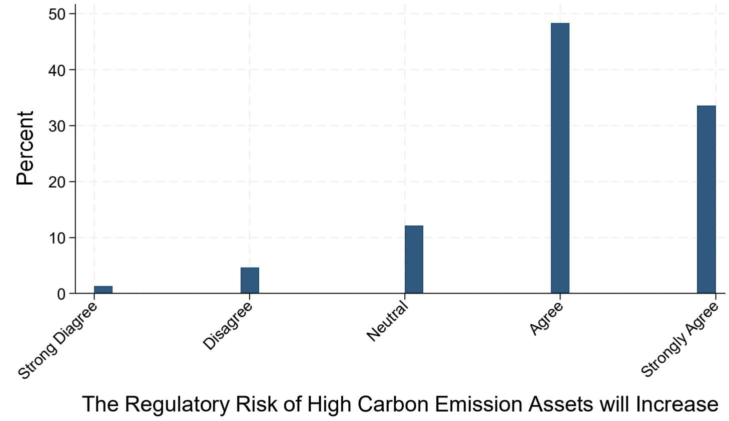

Fig S10. Distribution of responses "The regulatory risk of high carbon emission assets will increase"

Supplementary Information: Optimistic framing increases responsible investment of investment professionals

Panel Regression Analysis

Panel regression analysis is applied to more carefully examine the relationship between the framing treatments and the individual participant's optimistic orientation. We measure the influence of the Optimism frame (and other) treatments, and then combine this influence with their level of optimism as measured by the LOT-R instrument.

To measure the influence of the treatments, we compare the responsible investment choices of each individual with the average choices for each scenario. Naturally, there will be different preferences under each scenario. For example, there should be higher responsible preferences if there are lower ESG charges. However, we are particularly concerned with how this pattern of preferences is affected by the communication frame. We therefore set the difference between an individual's preferences and the average preferences for each scenario as the dependent variable. This variable is then modelled using an indicator variable for each treatment, control variables (eg age and female) and an optimism indicator variable.

The individual's measure of optimism is transformed into an indicator variable where one is the value for those with above average optimism and zero is the value for those with below average optimism. By combining this indicator variable with the indicator representing the optimism treatment, we can discern the marginal effect from these two dimensions – i.e., optimistic investors responding to an optimistic message.

A panel regression is an appropriate structure because all individual participants are exposed to the full range of scenarios (similar to a time series data set) with each scenario containing different ESG charges and volatilities. The panel therefore consists of individual participants by the six scenarios. The three treatments are represented by indicator variables (contrasting with the Control condition) and the interaction term will show the effect of optimistic individuals acting under the Optimism frame treatment. Control variables reflect the varying ages and which individuals were female. The regression model therefore takes the form:

Supplementary Information: Optimistic framing increases responsible investment of investment professionals

$$(S_{ijk} - C_j) = a_0 + a_5 Norm + a_6 Msg + a_7 Opt + a_8 Age_i + a_9 Female_i + a_1 ESG\_Con_j + a_2 ESG\_Bal_j + a_3 \left(Vol_{ESGCon_j} - Vol_{Con_j}\right) + a_4 \left(Vol_{ESGBal_j} - Vol_{Bal_j}\right) + a_{10} Optimistic_i + a_{11} Norm_i \times Optimistic_i + a_{12} Msg_i \times Optimistic_i + a_{13} Opt_i \times Optimistic_i$$

Where

S_{ijk} = Proportion sustainably allocated by participant i under scenario j in treatment k

C_j = Average proportion sustainably allocated by participants under scenario j in control treatment

a_0 = constant and a_1 to a_{13} = coefficients for variables

$Norm, Msg$ and Opt = Indictor variables for Norms, Messenger and Optimism treatments

Age_i and $Female_i$ = control variables for age and female

ESG_Con_j = ESG charge for conservative option in scenario j

ESG_Bal_j = ESG charge for balanced option in scenario j

$Vol\_ESG\_Con_j$ = Volatility of conservative option in scenario j

Vol_Con_j = Volatility of conservative option in scenario j

$Vol\_ESG\_Bal_j$ = Volatility of balanced option in scenario j

Vol_Bal_j = Volatility of balanced option in scenario j

$Optimistic_i$ = Indicator variable for above medium optimism score

$Norm_i \times Optimistic_i$ = interactive variable for the Norms treatment and the optimistic indicator variable

$Msg_i \times Optimistic_i$ = interactive variable for the Messenger treatment and the optimistic indicator variable

$Opt_i \times Optimistic_i$ = interactive variable for the Optimism treatment and the optimistic indicator variable

The interaction between optimism and an optimistic message is the most prominent finding from the panel regression. The results in Table 2 show a significant interaction effect between the optimistic individuals and the Optimism treatment – i.e., for the variable Opt_i x $Optimistic_i$.

VARIABLES	(1)<br>ESG allocation vs average for scenario
Norms	4.398
	(8.531)
Optimism	-7.166
	(6.092)
Messenger	-12.08
	(7.835)
Age	0.172
	(0.124)
Female	2.728
	(2.648)
Conventional option ESG charge	-0.682
	(0.929)
Balanced option ESG charge	0.235
	(0.829)

Conventional option risk difference	-2.776
	(2.585)
Conventional option risk difference	1.385
	(1.573)
Optimism	-3.216
	(5.895)
Norms x optimism	-6.954
	(10.69)
Optimism x optimism	17.70**
	(7.472)
Messenger x optimism	16.27*
	(9.231)
Constant	-4.696
	(7.579)
Observations	2,010
Number of id	335

Table S2. Regression results for the effect of variables on percentage of ESG invested compared to the average invested for each scenario. Notes: *** p<0.01, ** p<0.05, * p<0.1

****

F. STATA Code

```
* treatment
* 0 = Control
* 1 = Norms
* 2 = Optimism
* 3 = Messenger

* Generate portfolio choice dummies
gen pc1 =0
gen pc2 =0
gen pc3 =0
gen pc4 =0
gen pc5 =0
gen pc6 =0

* reshape command Convert data from wide form to long form

reshape long pc , i(id) j(portfolio)

* Create treatment dummies

gen t1 = 0
replace t1=1 if treatment ==1
gen t2 = 0
replace t2= 1 if treatment ==2
gen t3 =0
replace t3= 1 if treatment ==3
```

Supplementary Information: Optimistic framing increases responsible investment of investment professionals

```
* Create pc dummies again (ommitting pc1)

rename pc pc2
replace pc2 =1 if portfolio ==2
gen pc3 =0
replace pc3 =1 if portfolio ==3
gen pc4 =0
replace pc4=1 if portfolio ==4
gen pc5 = 0
replace pc5 =1 if portfolio ==5
gen pc6 = 0
replace pc6= 1 if portfolio ==6

* Create ESG % total allocation
gen esg_pct = (.)
replace esg_pct = (pc1_esc_con + pc1_esc_bal) if portfolio==1
replace esg_pct = (pc2_esc_con + pc2_esc_bal) if portfolio==2
replace esg_pct = (pc3_esc_con + pc3_esc_bal) if portfolio==3
replace esg_pct = (pc4_esc_con + pc4_esc_bal) if portfolio==4
replace esg_pct = (pc5_esc_con + pc5_esc_bal) if portfolio==5
replace esg_pct = (pc6_esc_con + pc6_esc_bal) if portfolio==6


* Create ESG % Conservative allocation
gen esg_pct_con = (.)
replace esg_pct_con = pc1_esc_con if portfolio==1
replace esg_pct_con = pc2_esc_con if portfolio==2
replace esg_pct_con = pc3_esc_con if portfolio==3
replace esg_pct_con = pc4_esc_con if portfolio==4
replace esg_pct_con = pc5_esc_con if portfolio==5
replace esg_pct_con = pc6_esc_con if portfolio==6

* Create ESG % Balanced allocation
gen esg_pct_bal = (.)
replace esg_pct_bal = pc1_esc_con if portfolio==1
replace esg_pct_bal = pc2_esc_con if portfolio==2
replace esg_pct_bal = pc3_esc_con if portfolio==3
replace esg_pct_bal = pc4_esc_con if portfolio==4
replace esg_pct_bal = pc5_esc_con if portfolio==5
replace esg_pct_bal = pc6_esc_con if portfolio==6

* Dummy = 1 if YES to 1m, 1 yr, 10yrs "I do not plan to increase my share of ESG investments
personal portfolio"
gen intention = 0
replace intention = 1 if att02sq001=="Yes"
replace intention = 1 if att02sq002=="Yes"
replace intention = 1 if att02sq003=="Yes"
gen intention_1y =0
replace intention_1y = 1 if att02sq001=="Yes"
replace intention_1y = 1 if att02sq002=="Yes"

* Create organization dummies

encode demo03a, gen(org)
```

```
label values org .
order org, before (demo03a)
replace org = 1 if org==2 // Financial Planning Catagory
replace org = 2 if org==5 // Consulting & Education Catagory + Other
replace org = 2 if org==6 // Consulting & Education Catagory + Other
replace org = 2 if org==7 // Consulting & Education Catagory + Other
replace org = 2 if org==10 // Consulting & Education Catagory + Other
replace org = 2 if org==11 // Consulting & Education Catagory + Other
replace org = 2 if org==12 // Consulting & Education Catagory + Other

replace org = 3 if org==8 // Funds Mgt & Super & Private Investor
replace org = 3 if org==9 // Funds Mgt & Super & Private Investor
replace org = 3 if org==13 // Funds Mgt & Super & Private Investor
replace org = 3 if org==4 // Funds Mgt & Super & Private Investor


gen fp = 0
replace fp = 1 if org==1 // financial planning industry dummy

gen sp = 0
replace sp = 1 if org ==2 // service provider industry

gen fm=0
replace fm = 1 if org==3 // Funds Mgt industry

*************************************************************************

* Create variable for allocation to option A
gen a = (.)
replace a = pc1_fossil_con if portfolio==1
replace a = pc2_fossil_con if portfolio==2
replace a = pc3_fossil_con if portfolio==3
replace a = pc4_fossil_con if portfolio==4
replace a = pc5_fossil_con if portfolio==5
replace a = pc6_fossil_con if portfolio==6

* Create variable for allocation to option B
gen b = (.)
replace b = pc1_esc_con if portfolio==1
replace b = pc2_esc_con if portfolio==2
replace b = pc3_esc_con if portfolio==3
replace b = pc4_esc_con if portfolio==4
replace b = pc5_esc_con if portfolio==5
replace b = pc6_esc_con if portfolio==6
* Create variable for allocation to option C
gen c = (.)
replace c = pc1_fossil_bal if portfolio==1
replace c = pc2_fossil_bal if portfolio==2
replace c = pc3_fossil_bal if portfolio==3
replace c = pc4_fossil_bal if portfolio==4
replace c = pc5_fossil_bal if portfolio==5
replace c = pc6_fossil_bal if portfolio==6
* Create variable for allocation to option D
gen d = (.)
replace d = pc1_esc_bal if portfolio==1
```

```
replace d = pc2_esc_bal if portfolio==2
replace d = pc3_esc_bal if portfolio==3
replace d = pc4_esc_bal if portfolio==4
replace d = pc5_esc_bal if portfolio==5
replace d = pc6_esc_bal if portfolio==6

* Naive Diversification between conditions

gen naive_diversification=(0)

replace naive_diversification=1 if a == 25 & b == 25 & c == 25 & c ==25

graph bar naive_diversification, over(treatment, label(labsize(large)) ///
relabel(1 "Control" 2 "Norms" 3 "Optimism" 4 "Messenger")) ///
blabel(bar, format(%4.2f) size(large)) ///
ytitle("Naive Diversification (Proportion)", size(large)) ///
yscale(range(0 1)) ylabel(0.2 0.4 0.6 0.8 1) ///
graphregion(color(white)) bar(1, fcolor(gray 9))

* Reverse code item 3 in Optimism Score

replace op01sq003 = 1 if op01sq003==5
replace op01sq003 = 2 if op01sq003==4
replace op01sq003 = 4 if op01sq003==2
replace op01sq003 = 5 if op01sq003==1

* Generate Aggregate Optimism Score from 3 items

gen optscore = (.)
replace optscore = op01sq001 + op01sq002 + op01sq003
summarize optscore

* Create dummy for optimism ******************

gen optimism = 1
replace optimism = 0 if optscore<11

* rename variable

rename demo02 age

* Create female dummy variable from str variable

encode demo01, gen(female)
label values female .
replace female = 0 if female==2

* Create variable for total % in Conservative allocation

gen con_pct = a + b

* Create variable for total % in Balanced allocation

gen bal_pct = c + d
```

Supplementary Information: Optimistic framing increases responsible investment of investment professionals

```
order pc2 pc3 pc4 pc5 pc6 esg_pct con_pct bal_pct a b c d t1 t2 t3 female demo01, before (condition)

* Create Interaction Variables

gen t1age = age*t1
gen t2age = age*t2
gen t3age = age*t3

gen t1optimism =t1*optimism
gen t2optimism =t2*optimism
gen t3optimism =t3*optimism

*gen t1optscore =t1*optscore
*gen t2optscore =t2*optscore
*gen t3optscore =t3*optscore

gen t1fp = t1*fp
gen t2fp = t2*fp
gen t3fp = t3*fp

gen t1sp = t1*fp
gen t2sp = t2*fp
gen t3sp = t3*fp

gen t1fm = t1*fm
gen t2fm = t2*fm
gen t3fm = t3*fm

* pc and treatment interactions
gen pc2t1 = pc2*t1
gen pc3t1 = pc3*t1
gen pc4t1 = pc4*t1
gen pc5t1 = pc5*t1
gen pc6t1 = pc6*t1

gen pc2t2 = pc2*t2
gen pc3t2 = pc3*t2
gen pc4t2 = pc4*t2
gen pc5t2 = pc5*t2
gen pc6t2 = pc6*t2

gen pc2t3 = pc2*t3
gen pc3t3 = pc3*t3
gen pc4t3 = pc4*t3
gen pc5t3 = pc5*t3
gen pc6t3 = pc6*t3


* Dummy if agree to "the environment is important to me"
gen important = (.)
replace important = 1 if att01sq002>3
replace important = 0 if att01sq002<4


* Dummy = 0 if YES to "I do not plan to increase my share of ESG investments ///
```

```
* personal portfolio - In ONE Month"
gen personalportfolio1m = (.)
replace personalportfolio1m = 1 if att02sq001=="Yes"
replace personalportfolio1m = 0 if att02sq001=="No"
count if personalportfolio1m == 1

count if personalportfolio1m == 1 & treatment ==0
count if personalportfolio1m == 1 & treatment ==1
count if personalportfolio1m == 1 & treatment ==2
count if personalportfolio1m == 1 & treatment ==3

******Summary Statistics Mean sd************

summarize esg_pct if intention==1 & org!=2 & treatment==0
summarize esg_pct if intention==1 & org!=2 & treatment==1
summarize esg_pct if intention==1 & org!=2 & treatment==2
summarize esg_pct if intention==1 & org!=2 & treatment==3

* Optimists
summarize esg_pct if intention==1 & org!=2 & optimism==1 & treatment==0
summarize esg_pct if intention==1 & org!=2 & optimism==1 & treatment==1
summarize esg_pct if intention==1 & org!=2 & optimism==1 & treatment==2
summarize esg_pct if intention==1 & org!=2 & optimism==1 & treatment==3

* Pessimists
summarize esg_pct if intention==1 & org!=2 & optimism==0 & treatment==0
summarize esg_pct if intention==1 & org!=2 & optimism==0 & treatment==1
summarize esg_pct if intention==1 & org!=2 & optimism==0 & treatment==2
summarize esg_pct if intention==1 & org!=2 & optimism==0 & treatment==3

* t-tests******************* all portfolios************************

gen esgpct_control = esg_pct if treatment==0 & intention==1 & org!=2
gen esgpct_norms = esg_pct if treatment==1 & intention==1 & org!=2
gen esgpct_optimism = esg_pct if treatment==2 & intention==1 & org!=2
gen esgpct_messenger = esg_pct if treatment==3 & intention==1 & org!=2


*robustness check, comment out relevant exclusion criteria when executing
*gen esgpct_control = esg_pct if treatment==0  & org!=2 /* & intention==1 */
*gen esgpct_norms = esg_pct if treatment==1  & org!=2 /* & intention==1 */
*gen esgpct_optimism = esg_pct if treatment==2   & org!=2  /* & intention==1 */
*gen esgpct_messenger = esg_pct if treatment==3  & org!=2 /*& intention==1 */

ttest esgpct_control = esgpct_norms, unpaired unequal
ttest esgpct_control = esgpct_optimism, unpaired unequal
ttest esgpct_control = esgpct_messenger, unpaired unequal

* test esgpct_optimism and optimimists esgpct_optimism

gen esgpct_optimism_opt = esgpct_optimism if optimism==1
ttest esgpct_optimism_opt = esgpct_optimism, unpaired unequal

* test optimists in OPT Treatment compared to optimists in Control treatment
gen esgpct_control_opt = esgpct_control if optimism==1
```

```
ttest esgpct_optimism_opt = esgpct_control_opt, unpaired unequal

* test optimists in NORMS Treatment compared to optimists in Control treatment
gen esgpct_norms_opt = esgpct_norms if optimism==1
ttest esgpct_norms_opt = esgpct_control_opt, unpaired unequal

* test optimists in MESSENGER Treatment compared to optimists in Control treatment
gen esgpct_messenger_opt = esgpct_messenger if optimism==1
ttest esgpct_messenger_opt = esgpct_control_opt, unpaired unequal

* test optimists in OPT Treatment compared to Pessimists in OPT treatment
gen esgpct_optimism_pessimist = esgpct_optimism if optimism==0
ttest esgpct_optimism_opt =  esgpct_optimism_pessimist, unpaired unequal

* test optimists in Messenger Treatment compared to Pessimists in Messenger treatment
gen esgpct_messenger_pessimist = esgpct_optimism if optimism==0
ttest esgpct_messenger_opt = esgpct_messenger_pessimist, unpaired unequal

* test optimists in Norms Treatment compared to Pessimists in Norms treatment
gen esgpct_norms_pessimist = esgpct_norms if optimism==0
ttest esgpct_norms_opt = esgpct_norms_pessimist, unpaired unequal

* test optimists in Control Treatment compared to Pessimists in Control treatment
gen esgpct_control_pessimist = esgpct_control if optimism==0
ttest esgpct_control_opt = esgpct_control_pessimist, unpaired unequal

*** t-tests conservative (b) and balanced options separately (d)*****

gen esgcon_control = b if treatment==0 & intention==1 & org!=2
gen esgcon_norms = b if treatment==1 & intention==1 & org!=2
gen esgcon_optimism = b if treatment==2 & intention==1 & org!=2
gen esgcon_messenger = b if treatment==3 & intention==1 & org!=2

gen esgbal_control = d if treatment==0 & intention==1 & org!=2
gen esgbal_norms = d if treatment==1 & intention==1 & org!=2
gen esgbal_optimism = d if treatment==2 & intention==1 & org!=2
gen esgbal_messenger = d if treatment==3 & intention==1 & org!=2

ttest esgcon_control = esgcon_norms, unpaired unequal
ttest esgcon_control = esgcon_optimism, unpaired unequal
ttest esgcon_control = esgcon_messenger, unpaired unequal

ttest esgbal_control = esgbal_norms, unpaired unequal
ttest esgbal_control = esgbal_optimism, unpaired unequal
ttest esgbal_control = esgbal_messenger, unpaired unequal

sum b if treatment==0 & intention==1 & org!=2
sum b if treatment==1 & intention==1 & org!=2
sum b if treatment==2 & intention==1 & org!=2
sum b if treatment==3 & intention==1 & org!=2

sum d if treatment==0 & intention==1 & org!=2
sum d if treatment==1 & intention==1 & org!=2
sum d if treatment==2 & intention==1 & org!=2
sum d if treatment==3 & intention==1 & org!=2
```

Supplementary Information: Optimistic framing increases responsible investment of investment professionals

```
**** Robustness check 50% randomly drawn sample *

*sample 50 if treatment == 2
*graph bar (mean) esg_pct if intention==1 & org!=2, ytitle("ESG Invested %", size(large)) ///
*over(treatment, label(labsize(large)) relabel(1 "Control" 2 "Norms" 3 "Optimism" 4 "Messenger"))
///
*yscale(range(0 100)) ylabel(20 40 60 80 100) graphregion(color(white)) ///
*blabel(bar, format(%4.2f) size(large)) bar(1, fcolor(gray))

********************Effect size************************************

esize unpaired esgpct_optimism ==  esgpct_control  //Cohen's d 0.11
esize unpaired esgpct_optimism_opt == esgpct_control_opt //Cohoen's d 0.32
esize unpaired esgpct_optimism_opt == esgpct_optimism_pessimist //Cohoen's d 0.46
esize unpaired esgpct_messenger_opt == esgpct_messenger_pessimist //Cohoen's d 0.29
esize unpaired esgpct_norms_opt == esgpct_norms_pessimist //Cohoen's d 0.27
esize unpaired esgbal_control ==  esgbal_optimism
esize unpaired esgcon_control ==  esgcon_optimism

***************************** GRAPHS *****************************

* Fig 1 Graph %ESG by 6 Portfolios ***
graph bar (mean) esg_pct if intention==1 & org!=2, ytitle("ESG Invested %", size(large)) ///
over(portfolio) over(treatment, label(labsize(large)) ///
relabel(1 "Control" 2 "Norms" 3 "Optimism" 4 "Messenger")) ///
yscale(range(0 100)) ylabel(20 40 60 80 100) ///
graphregion(color(white)) bar(1, fcolor(gray 9))

* Fig S1 ESG% by Condition

graph bar (mean) esg_pct if intention==1 & org!=2, ytitle("ESG Invested %", size(large)) ///
over(treatment, label(labsize(large)) relabel(1 "Control" 2 "Norms" 3 "Optimism" 4 "Messenger")) ///
yscale(range(0 100)) ylabel(20 40 60 80 100) graphregion(color(white)) ///
blabel(bar, format(%4.2f) size(large)) bar(1, fcolor(gray))

* ESG% by Condition, Optimists

graph bar (mean) esg_pct if intention==1 & org!=2 & optimism==1 , ytitle("ESG Invested %",
size(large)) ///
over(treatment, label(labsize(large)) relabel(1 "Control" 2 "Norms" 3 "Optimism" 4 "Messenger")) ///
yscale(range(0 100)) ylabel(20 40 60 80 100) graphregion(color(white)) ///
blabel(bar, format(%4.2f) size(large)) bar(1, fcolor(gray))
graph export graph_esg_condition_optimists.png, replace

* Graph %ESG by 6 Portfolios for CONSERVATIVE

graph bar (mean) b if intention==1 & org!=2, ///
ytitle("ESG Invested %", size(large)) over(portfolio) ///
over(treatment, label(labsize(large)) ///
relabel(1 "Control" 2 "Norms" 3 "Optimism" 4 "Messenger")) ///
graphregion(color(white)) yscale(range(0 60)) ///
ylabel(10 20 30 40 50 60) bar(1, fcolor(gray 9))

* Graph %ESG by 6 Portfolios for BALANCED
```

```
graph bar (mean) d if intention==1 & org!=2, ///
ytitle("ESG Invested %", size(large)) over(portfolio) ///
over(treatment, label(labsize(large)) ///
relabel(1 "Control" 2 "Norms" 3 "Optimism" 4 "Messenger")) ///
graphregion(color(white)) yscale(range(0 60)) ///
ylabel(10 20 30 40 50 60)  bar(1, fcolor(gray 9))

* Graphs of questionnaire responses
histogram optscore, percent xlabel(#15)

histogram att01sq001, percent color(navy) xlabel(1 "Strong Diagree" ///
2 "Disagree" 3 "Neutral" 4 "Agree" 5 "Strongly Agree", angle(forty_five) ) ///
xtitle("I Expect the Price of ESG Investments to Increase Over Time")

histogram att01sq002, percent color(navy) xlabel(1 "Strongly Disagree" ///
2 "Disagree" 3 "Neutral" 4 "Agree" 5 "Strongly Agree", angle(forty_five) )  yscale(range(0 100)) ///
ylabel(20 40 60 80 100) xtitle("The Environment is Important to Me")

histogram att01sq003, percent color(navy) xlabel(1 "Strongly Disagree" ///
2 "Disagree" 3 "Neutral" 4 "Agree" 5 "Strongly Agree", angle(forty_five) ) ///
xtitle("I Put a Lot of Thought into my Investment Decisions")

histogram att01sq004, percent color(navy) xlabel(1 "Strongly Disagree" ///
2 "Disagree" 3 "Neutral" 4 "Agree" 5 "Strongly Agree", angle(forty_five) ) ///
xtitle("The Regulatory Risk of High Carbon Emission Assets is High")

histogram att01sq005, percent color(navy) xlabel(1 "Strong Diagree" ///
2 "Disagree" 3 "Neutral" 4 "Agree" 5 "Strongly Agree", angle(forty_five) ) ///
xtitle("The Regulatory Risk of High Carbon Emission Assets will Increase")

****************************************
* Balance table

* Average Age
egen meanage=mean(age)

* Average Age
summarize age if treatment==0
summarize age if treatment==1
summarize age if treatment==2
summarize age if treatment==3

* optscore
summarize optscore if treatment==0
summarize optscore if treatment==1
summarize optscore if treatment==2
summarize optscore if treatment==3

*Average experience demo04
summarize demo04 if treatment==0
summarize demo04 if treatment==1
summarize demo04 if treatment==2
summarize demo04 if treatment==3
```

Supplementary Information: Optimistic framing increases responsible investment of investment professionals

```
***************************REGRESSIONS**************************

* Random Effects Regression

sort portfolio
merge portfolio using ave_esg_pct.dta
sort id portfolio treatment esg_pct bal_pct female demo01
drop _merge
generate esg_pct_diff = esg_pct - ave_esg_pct

*label variables
label variable t1 "Norms"
label variable t2 "Optimism"
label variable t3 "Messenger"
label variable age "Age"
label variable female "Female"
label variable cons_esg_chg "Conventional option ESG charge"
label variable bal_esg_chg "Balanced option ESG charge"
label variable cons_esg_vol_diff "Conventional option risk difference"
label variable bal_esg_vol_diff "Conventional option risk difference"
label variable optimism "Optimism"
label variable t1optimism "Norms x optimism"
label variable t2optimism "Optimism x optimism"
label variable t3optimism "Messenger x optimism"
label variable esg_pct_diff "ESG allocation vs average for scenario"
tsset id portfolio

xtreg esg_pct_diff t1 t2 t3 age female cons_esg_chg bal_esg_chg cons_esg_vol_diff bal_esg_vol_diff
///
optimism t1optimism t2optimism t3optimism if intention==1 & org!=2, robust cluster(id)
outreg2 using esg_pct_diff.doc, keep(t1 t2 t3 age female cons_esg_chg bal_esg_chg
cons_esg_vol_diff ///
bal_esg_vol_diff optimism t1optimism t2optimism t3optimism) replace title(Regression results) ///
label
```
